# Exploratory Study of the Urine Protein-to-Creatinine Ratio in Apparently Healthy Horses

**DOI:** 10.3390/vetsci12080783

**Published:** 2025-08-21

**Authors:** Simona Kovarikova, Jana Blahova, Veronika Steffenova, Natalie Vaskova, Petr Jahn

**Affiliations:** 1 Department of Animal Protection and Welfare and Veterinary Public Health, Faculty of Veterinary Hygiene and Ecology, University of Veterinary Sciences, 61242 Brno, Czech Republic; blahovaj@vfu.cz (J.B.);; 2 Equine Clinic, Faculty of Veterinary Medicine, University of Veterinary Sciences, 61242 Brno, Czech Republic; jahnp@vfu.cz

**Keywords:** proteinuria, renal disease, biomarkers

## Abstract

Currently, there is relatively little published information on the urine protein-to-creatinine (UPC) ratio in horses, a parameter commonly used in dogs and cats. The aim of this study was to determine the reference range of UPC in urine samples of apparently healthy horses and to assess the influence of age and sex. Urinalysis and the determination of urinary protein and creatinine concentrations and ratios were performed. A total of 118 urine samples from apparently healthy horses were included in the study: 10 from foals younger than 6 months and 108 from horses older than 1 year. The median UPC for foals younger than 6 months and horses aged 1–4 years, 5–10 years, 11–17 years and older than 18 years were 0.074, 0.073, 0.070, 0.083, and 0.070, respectively. Using the non-parametric Kruskal–Wallis test, statistically significant differences were found between the youngest age group and both groups of adult horses (i.e., 5–10 years and 11–17 years); however, these differences were not confirmed by the General Linear Model, likely due to the small sample sizes in some categories. There was no effect of sex on UPC. The indicative reference range was set for the group of horses aged 5–17 years as 0.02–0.18.

## Simple Summary

Urinalysis should be a routine part of the renal diagnostic evaluation in all patients. One of the parameters determined is the urine protein-to-creatinine ratio, which is commonly used in dogs and cats. However, information from horses on this parameter is relatively sparse. Thus, the aim of this study was to establish a reference range for the urinary protein-to-creatinine ratio in apparently healthy horses and to assess the effect of age and sex on this parameter. A statistically significant effect of age but not sex was found. The indicative reference range was set for horses aged 5–17 years as 0.02–0.18. This range can be used for comparison when assessing kidney function in horses with a health problem.

## 1. Introduction

The evaluation of urine is an integral component of the diagnostic process in clinical practice as it provides insights into urinary system function. Given that the kidneys play a crucial role in maintaining homeostasis, urinalysis can yield information regarding the overall metabolic state [1]. One of the advantages of urine examination is the ability to obtain a sample in a non-invasive manner, as well as the potential to identify certain issues earlier than through blood tests (e.g., in the diagnosis of acute kidney injury) [2,3]. One of the parameters assessed in urinalysis is the determination of protein concentration. This can be evaluated through the reaction on a dipstick, using a sulfosalicylic acid test, or by measuring the urinary protein-to-creatinine (UPC) ratio. Unfortunately, dipsticks are often not very reliable in veterinary medicine, and the sulfosalicylic acid test is only semi-quantitative, being influenced by alkaline urine pH, which can lead to false negative results. The measurement of the UPC has become the gold standard for assessing proteinuria in dogs and cats [4]. Currently, it is also being established in other domestic livestock and pet animals [5,6,7,8,9], as well as in wild animals kept in captivity [10,11,12,13].

In comparison to dogs and cats, there is limited information regarding the use of the UPC in horses. Uberti et al. (2009) [14] demonstrated that the UPC is a reliable method for estimating urine protein excretion in horses. However, most data on UPC in horses come from a small number of horses, specifically defined groups, or case reports [15,16,17]. This study aims to establish reference ranges for UPC in horses and to assess the influence of age and sex on this parameter.

## 2. Materials and Methods

Urine samples from apparently healthy horses were included in this study. In all cases, the horses belonged to private owners who were approached and asked to participate in the study. Horses were established as being healthy through medical history (no health issues or medication), a complete physical examination, and a normal body condition score. Urine samples were collected using spontaneous voiding and only in connection with normal physical activity (no strenuous exercise for at least 48 h before sampling). Horses were excluded if they were deemed unhealthy or had abnormal results of physical examination, had been vaccinated in the preceding 30 days, received medications other than routine antiparasitic agents, or were pregnant or lactating mares. The presence of blood or more than five leukocytes per high-power field in the urine sample was another criterion for exclusion from the study. Consent for urine collection and further analysis was obtained from the owners.

Immediately after collection, basic urine examination was performed, which included assessing physical properties (color, turbidity, and determining density using a handheld refractometer—RUR2-ATC, Bellingham + Stanley, Tunbridge Wells, UK), chemical properties determined with the help of a dipstick (HeptaPHAN, Erba Lachema, Brno, Czech Republic), and microscopic examination. The dipstick was immersed in the urine sample for 1–2 s according to the manufacturer’s instructions, and the resulting color reaction was then compared with the supplied scale after 60 s. The results on the dipstick were evaluated simultaneously by two people. The dipstick was used to evaluate pH (range 5–9) with an accuracy of half a degree; the presence of protein (evaluated reaction: 0 ~ negative, 1+ ~ 30 mg/dL; 2+ ~ 100 mg/dL; 3+ ~ 500 mg/dL); the presence of glucose (evaluated reaction: 0 ~ negative; 1+ ~ 50 mg/dL, 2+ ~ 100 mg/dL; 3+ ~ 300 mg/dL; 4+ ~ 1000 mg/dL); ketone bodies (0 ~ negative; 1+ ~ 16 mg/dL; 2+ ~ 52 mg/dL; 3+ ~ 156 mg/dL); urobilinogen (0 ~ negative; 1+ ~ 1 mg/dL; 2+ ~ 3 mg/dL; 3+ ~ 6 mg/dL; 4+ ~ 12 mg/dL); bilirubin (0 ~ negative; 1+; 2+ and 3+ arbitrary units); and blood (0 ~ negative; 1+ ~ 5–10 erythrocytes/μL; 2+ ~ 50 erythrocytes/μL; 3+ ~ 250 erythrocytes/μL). Cases where the reaction was evaluated as 0.5+ were classified in the 1+ reaction group for simplicity. The urine sample was then frozen and stored at −20 °C until laboratory analysis, which was conducted no later than 4 weeks after collection.

After thawing, the urine sample was centrifuged (3000 rpm for 3 min), and the supernatant was used for laboratory analysis. The protein concentration in the urine was determined using commercial kits and a colorimetric method (protein reaction with pyrogallol red and molybdate (total protein–urine, liquor; Biovendor, Brno, Czech Republic, with detection limit 20 mg/L).

The creatinine concentration in urine was measured using the modified Jaffe method (Creatinine, Biovendor, Brno, Czech Republic). Before analysis, urine samples were diluted with 50 μL of sample and 2450 μL of ultrapure water. All tests were performed on the Konelab 20i automated analyzer (ThermoFisher Scientific, Waltham, MA, USA). The ratio of protein to creatinine concentration in urine was then calculated from the results.

Horses were divided into groups according to age: foals up to 6 months and horses aged 1–4 years, 5–10 years, 11–17 years, and older than 18 years. For horses older than 1 year, the results were also compared between males and females. Data were analyzed using Unistat for Excel, version 6.5. The normality of data distribution was assessed using the Shapiro–Wilk test, and homogeneity of variances was tested using Levene’s test. If both assumptions were met, differences between age categories were evaluated using one-way ANOVA followed by Tukey’s HSD post hoc test. When the assumptions of normality or homogeneity were not satisfied, a non-parametric Kruskal–Wallis test was applied. In addition, a General Linear Model (GLM) was also used for UPC to compare age categories, with age group as a fixed factor. We applied data transformation (e.g., logarithmic) to improve normality of UPC values within age groups before fitting the GLM. While the transformation improved data distribution in most groups, one group still did not fully meet the normality assumption. Despite this limitation, the GLM was applied. Due to the partial violation of assumptions and small sample sizes, the GLM results should be interpreted cautiously and considered complementary to the non-parametric Kruskal–Wallis test, which remains the primary method for assessing group differences in this study. Spearman’s rank correlation coefficient was used to calculate the correlation coefficients (r_s_). Differences were considered statistically significant at *p* < 0.05. Reference Value Advisor (for use in Microsoft Excel) was used to calculate the reference intervals.

## 3. Results

### 3.1. Animals

A total of 118 urine samples from apparently healthy horses were included in the study. Ten samples were collected from foals younger than 6 months, and 108 samples were from horses older than 1 year. In the foal group, there were 6 males and 4 females aged between 1 and 5 months (mean 3 months). All foals were of the Czech Warmblood breed. In the group of horses older than 1 year, there were 63 mares and 45 males (41 geldings and 4 stallions) aged between 1 and 26 years (mean 12.2 years). The breeds of horses older than one year are listed in Table 1. A more detailed characterization of all horses divided into groups is provided in Table 2.

### 3.2. Urinalysis

Urine color ranged from pale yellow to brown, and turbidity varied from clear to completely opaque. In foals, most samples were transparent (8; 80.0%), whereas in horses older than one year, the majority of samples (95; 88.0%) showed varying degrees of turbidity. A significantly lower (*p* < 0.05) urine specific gravity (USG) was found in foals compared to horses older than one year (median 1.010 and 1.038, respectively; ranges 1.004–1.030 and 1.007–1.060, respectively). The urine pH for all horses older than one year ranged from 7 to 9, while in foals it ranged from 6 to 9, with no statistically significant difference observed.

When evaluating the dipstick, negative reactions for the presence of glucose, ketone bodies, and blood were recorded in all cases. In foals, negative reactions were also noted for the presence of protein, urobilinogen, and bilirubin. In horses older than one year, there were negative reactions for protein in 43 cases (39.8%), a reaction of 1+ in 32 samples (29.6%), a reaction of 2+ in 24 cases (22.2%), and a reaction of 3+ in 9 cases (8.4%). The majority of horses older than one year had negative reactions for the presence of urobilinogen (82; 75.9%) and bilirubin (93; 86.1%). Details of urinalysis are presented in Table 3. Microscopic examination of the samples revealed the presence of crystals in all cases. These included calcium carbonate crystals (97; 82.2%), calcium oxalate (56; 51.9%), and calcium phosphate (13; 11.0%). The presence of crystals was the cause of turbidity, and the number of crystals in the sample corresponded to the degree of turbidity.

### 3.3. Laboratory Analysis

**Figure 1:**
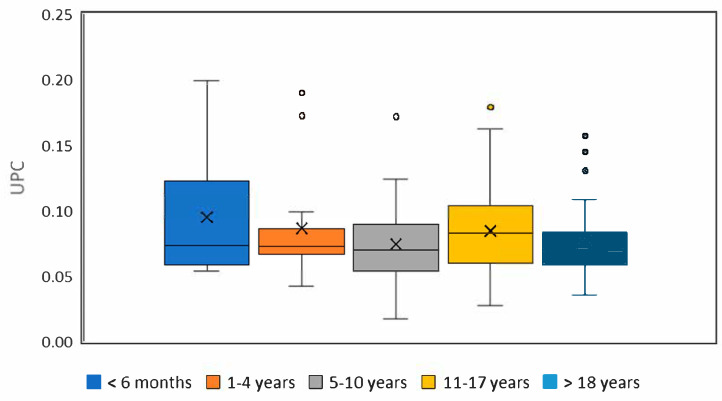
Urine protein-to-creatinine (UPC) ratio in groups of horses that were created according to age. Within each boxplot, the horizontal lines denote the median values, the cross indicates the mean value, the boxes extend from the 25th to the 75th percentile of the value distribution of each group, and the lower and upper whiskers indicate the smallest value within 1.5 times the interquartile range below the 25th percentile and the largest value within 1.5 times the interquartile range above the 75th percentile, respectively.

Statistically significant differences between individual age groups were found in urinary protein and creatinine concentrations. For the urine protein-to-creatinine (UPC) ratio, differences between age categories were evaluated using both the Kruskal–Wallis test and the General Linear Model (GLM). The Kruskal–Wallis test revealed significantly higher UPC values in both groups of adult horses (i.e., 5–10 years and 11–17 years) compared to the youngest group. The GLM, however, did not confirm these differences, most likely due to the limited sample sizes in some categories, which may reduce the statistical power of this method under such conditions. Therefore, the results of the Kruskal–Wallis test are considered more reliable for the current dataset. The detailed results comparing all age groups are shown in Table 4, and boxplots for UPC are presented in Figure 1. When comparing males and females over one year of age, there were no significant differences in urinary protein and creatinine concentrations and UPC values. A statistically significant positive correlation was found between urine specific gravity and the result of the urine protein test on the dipstick (r_s_ 0.23, *p* = 0.006) and a statistically significant negative correlation between the result of the protein test on the dipstick and UPC (r_s_ −0.22, *p* = 0.009) and between urine specific gravity and UPC (r_s_ −0.43, *p* = 0.000). Samples with a positive reaction to the presence of protein on the dipstick had a significantly higher specific gravity compared to samples with a negative reaction. However, no significant difference was found when comparing the UPC values between these two groups.

Due to the differences between the groups, only values from horses aged 5–17 years (68 horses) were used to calculate a reference range; the indicative reference range for this category of horses was set at 0.02–0.18. All data obtained from urinalysis and chemical analysis are listed in Supplementary Materials: Table S1.

## 4. Discussion

Urinalysis and UPC evaluation are standard tools for assessing the condition and function of the urinary tract in dogs and cats. Information on the use of this parameter in horses is rather scarce, so the aim of this study was to expand the knowledge of UPC in horses, to assess the influence of age and sex, and to establish a reference range for UPC in apparently healthy horses.

A total of 118 urine samples were included in the study, and a comprehensive examination was conducted. The results of the initial urine analysis align with previously published studies: equine urine is normally alkaline and often slightly turbid due to the presence of crystals, mainly calcium carbonate [18,19]. In our cohort, most samples (80%) from foals up to 6 months old were transparent, and fewer crystals were identified microscopically. This may be related to the significantly lower urine specific gravity compared to older horses over one year. According to published reports, newborn foals have yellow to amber urine with moderate turbidity and mild hypersthenuria. After nursing begins, urine turns pale yellow, clear, and watery [20]. In healthy foals, urine was generally dilute, with specific gravity reaching as high as 1.027; thus, a wide range of USG was observed [15]. Our cohort did not include any newborn foals but consisted of foals aged between one and five months. Therefore, our findings correspond with the characteristics of foal urine after nursing begins as described previously [15,20]. All urine samples collected from apparently healthy horses in our study tested negative for glucose, ketone bodies, and blood, and most were also negative for urobilinogen and bilirubin. This is again consistent with findings reported in healthy horses [18,20].

The amount of protein in the urine can be determined using a dipstick, a sulfosalicylic acid test, or by determining the urine protein-to-creatinine ratio. In practice, the use of dipsticks is the quickest and simplest, but this is associated with a relatively large error rate and many false positive samples, particularly in more alkaline and more concentrated urine samples [18,20]. Our results confirm this. We found a significant negative correlation between the reaction to the presence of protein on the dipstick and UPC. Samples that reacted positively to the presence of protein on the dipstick had a higher specific gravity than samples with a negative reaction. In a subsequent comparison, no difference in UPC values was found between samples with positive and negative reactions to the presence of protein on the dipstick. Thus, urine dipsticks are not a suitable tool for determining the amount of protein in equine urine. The relationship between the result of the protein reaction on the dipstick, the specific gravity, and the UPC and possibly other variables in the urine of horses could be addressed in more detail in a future study, ideally on a larger and more stratified cohort and possibly using samples from horses with some health problem.

The UPC determination is considered the gold standard for the quantification of proteinuria in dogs and cats. In horses, it is a reliable method for estimating 24 h protein excretion. For horses, a reference range of 0.0–0.37 has been suggested, but this work is based on examination of 11 female horses and 6 female ponies only [14]. Therefore, we wanted to increase the number of samples examined and assess the effect of age and gender. Urine samples were also always collected at least 48 h after strenuous physical activity of the horses as the activity can lead to significant proteinuria [21].

No statistically significant differences were found when comparing results from males and females. When comparing groups formed by age, lower urinary protein and creatinine concentrations are evident in foals younger than 1 year. This fact may have several explanations: The urine specific gravity of foals and thus the concentration of substances contained in it is lower compared to older horses. The lower concentration of creatinine in the urine of foals may also be related to the smaller amount of muscle mass and the lower concentration of creatinine in the blood. A similar finding was found in guinea pig pups [8].

The UPC values found in horses are similar to those reported for healthy dogs and cats [4]. Although statistically significant differences (*p* < 0.05) were found between groups created by age, they are likely to be of no clinical significance due to their small magnitude.

Our results are lower than those suggested by Uberti et al. (2009) [14]. It is possible that this difference could be caused by a different method of measuring protein concentration in urine. Currently, three methods are used to measure urine protein concentration in animals: turbidimetric using benzethonium chloride and two colorimetric methods using either pyrogallol red or Coomassie brilliant blue. None of these methods is considered a reference method, and all the methods are used in scientific studies. In Uberti’s study, they used a method based on the color change of Coomassie brilliant blue in response to various concentrations of protein [14]. In our previous study, we compared the turbidimetric and colorimetric methods using pyrogallol red to measure protein concentration in urine in dogs, cats, horses, and guinea pigs. In horses, the Passing–Bablok analysis found a significant constant and proportional difference, and the Bland–Altman method found a significant bias between the two methods [22]. This clearly shows that the method used to measure protein concentration in urine plays a role and thus also influences the UPC result. It should be noted that our results are valid for the colorimetric method using pyrogallol red.

A limitation of this study is that hematological and biochemical blood tests were not performed, which could have revealed a subclinical health issue that could affect renal function and possibly lead to proteinuria. Given the differences found in foals younger than 6 months, it would be prudent to focus on this age group and repeat urinalysis in a larger number of individuals. We are also aware that due to differences in UPC between groups of horses created according to age, we do not have a sufficient number of samples to establish a definitive reference range for UPC in apparently healthy horses. For this reason, the range we propose is only indicative.

## 5. Conclusions

UPC values in apparently healthy horses are similar to those of healthy dogs and cats. No effect of sex was found. UPC values are influenced by age; for horses aged 5–17 years, we suggest an indicative range of 0.02–0.18.

## Figures and Tables

**Table 1:** Breeds of horses over 1 year of age represented in the study.

Breed	Number
Czech Warmblood	47
Kladruber	18
Arabian horse	15
Haflinger	7
Pony	5
Slovak Warmblood	3
American Quarterhorse	2
Bohemian-Moravian Belgian Horse	2
Dutch Warmblood	2
Coldblood	1
Irish Cob	1
Polish Warmblood	1
Oldenburg Horse	1
Brabant Horse	1
American Paint Horse	1
Welsh Cob	1
**Total**	**108**

**Table 2:** Detailed characteristics of the groups formed according to the age of the horses.

Age Category	≤6 Months	1–4 Years	5–10 Years	11–17 Years	≥18 Years
*n*	10	14	33	35	26
Females (mares)	4	10	22	22	9
Males	6 (all stallions)	4 (all geldings)	11 (all geldings)	13 (12 geldings, 1 stallion)	17 (14 geldings, 3 stallions)

**Table 3:** Detailed results of urinalysis in foals and groups of horses over 1 year of age. Significant differences (*p* < 0.05) in specific gravity and pH among groups are indicated by different alphabetical superscripts.

Age Category	≤6 Months	1–4 Years	5–10 Years	11–17 Years	≥18 Years
*n*	10	14	33	35	26
**Transparency**	8 (80%)	3 (21.4%)	2 (6.1%)	5 (14.3%)	3 (11.5%)
**Turbidity**	2 (20%)	11 (78.6%)	31 (93.9%)	30 (85.7%)	23 (88.5%)
**Specific gravity**					
Median	1.010 ^a^	1.034 ^b^	1.041 ^b^	1.038 ^b^	1.037 ^b^
Range	1.004–1.030	1.007–1.057	1.025–1.060	1.008–1.058	1.020–1.047
**pH**					
Median	8.0 ^a^	8.0 ^a^	8.5 ^a^	8.5 ^a^	8.5 ^a^
Range	6.0–9.0	7.5–9.0	7.0–9.0	7.0–9.0	7.0–9.0
**Protein**					
0+	10 (100%)	7 (50.0%)	15 (45.5%)	16 (45.7%)	5 (19.2%)
1+	0	4 (28.6%)	8 (24.2%)	11 (31.4%)	9 (34.6%)
2+	0	2 (14.3%)	6 (18.2%)	5 (14.3%)	11 (42.3%)
3+	0	1 (7.1%)	4 (12.1%)	3 (8.6%)	1 (3.9%)
**Urobilinogen**					
0+	10 (100%)	11 (78.6%)	26 (78.8%)	28 (77.1%)	20 (76.9%)
1+	0	3 (21.4%)	7 (21.2%)	8 (22.9%)	6 (23.1%)
**Bilirubin**					
0+	10 (100%)	13 (92.9%)	28 (84.8%)	32 (91.4%)	20 (76.9%)
1+	0	1 (7.1%)	4 (12.1%)	3 (8.6%)	6 (23.1%)
2+	0	0	1 (3.0%)	0	0

The dipstick was used to evaluate pH (range 5–9) with an accuracy of half a degree; the presence of protein (evaluated reaction: 0 ~ negative, 1+ ~ 30 mg/dL; 2+ ~ 100 mg/dL; 3+ ~ 500 mg/dL); the presence of glucose (evaluated reaction: 0 ~ negative; 1+ ~ 50 mg/dL, 2+ ~ 100 mg/dL; 3+ ~ 300 mg/dL; 4+ ~ 1000 mg/dL); ketone bodies (0 ~ negative; 1+ ~ 16 mg/dL; 2+ ~ 52 mg/dL; 3+ ~ 156 mg/dL); urobilinogen (0 ~ negative; 1+ ~ 1mg/dL; 2+ ~ 3 mg/dL; 3+ ~ 6 mg/dL; 4+ ~ 12 mg/dL); bilirubin (0 ~ negative; 1+; 2+ and 3+ arbitrary units); and blood (0 ~ negative; 1+ ~ 5–10 erythrocytes/μL; 2+ ~ 50 erythrocytes/μL; 3+ ~ 250 erythrocytes/μL).

**Table 4:** Concentrations of urine protein and creatinine (UPC) and their ratios in foals and horses over 1 year of age. Statistical differences (*p* < 0.05, indicated by different letters) were evaluated based on data distribution. When the assumptions of normality and homogeneity of variances were met, one-way ANOVA was applied. When these assumptions were not satisfied, the non-parametric Kruskal–Wallis test was used.

Age Category	≤6 Months	1–4 Years	5–10 Years	11–17 Years	≥18 Years
*n*	10	14	33	35	26
**Urinary protein (mg/L)**					
Median	52.4 ^c^	181.8b ^c^	171.7 ^a^	157.3 ^ab^	150.9 ^b^
Range	21.4–561.8	50.2–248.9	136.5–220.5	25.3–243.7	75.0–235.2
95% CI	24.3–175.2	152.2–204.6	157.2–181.7	151.8–168.3	136.7–175.1
**Urinary creatinine (mmol/L)**					
Median	5.8 ^d^	20.9 ^bd^	22.1 ^a^	18.1 ^ac^	19.0 ^bc^
Range	2.6–25.0	2.6–35.8	7.9–72.6	2.1–57.3	4.1–44.1
95% CI	3.4–21.4	17.9–26.1	20.2–24.4	14.3–23.7	14.8–24.7
**UPC ratio**					
Median	0.074 ^b^	0.073 ^bc^	0.070 ^ac^	0.083 ^a^	0.070 ^bc^
Range	0.05–0.20	0.04–0.19	0.02–0.17	0.03–0.18	0.04–0.16
95% CI	0.06–0.14	0.06–0.10	0.06–0.08	0.06–0.09	0.06–0.08

CI—confidence interval.

## Data Availability

Data is contained within the Supplementary Materials.

## Supplementary Materials

The following supporting information can be downloaded at: https://www.mdpi.com/article/10.3390/vetsci12080783/s1, Table S1.

## Abbreviations

r_s_	Correlation coefficient
UPC	Urine protein-to-creatinine
GLM	General Linear Model
