# Supplementary material for: Exploratory Study of the Urine Protein-to-Creatinine Ratio in Apparently Healthy Horses
Source: Vet Sci. 2025 Aug 21;12(8):783. doi: 10.3390/vetsci12080783 (PMC12389935; doi:10.3390/vetsci12080783)
Supplement: Supplementary file 1 [file vetsci-12-00783-s001.zip › vetsci-3784082-supplementary.docx]

|  | **Breed** | **Age** | **Sex** | **USG** | **pH** | **Protein** | **Glucose** | **Ketones** | **Urobilinogen** | **Bilirubin** | **Blood** | **TP Biovendor mg/l** | **Creatinine (mmol/l)** | UPC |
| --- | --- | --- | --- | --- | --- | --- | --- | --- | --- | --- | --- | --- | --- | --- |
| **1** | CW | 24 | mare | 1.039 | 8 | 2 | 0 | 0 | 1 | 0 | 0 | 151.95 | 19.74 | **0.07** |
| **2** | CW | 3 | gelding | 1.038 | 9 | 3 | 0 | 0 | 1 | 0 | 0 | 183.80 | 19.79 | **0.08** |
| **3** | CW | 18 | gelding | 1.035 | 8 | 1 | 0 | 0 | 0 | 0 | 0 | 114.16 | 19.06 | **0.05** |
| **4** | CW | 6 | mare | 1.046 | 9 | 1 | 0 | 0 | 0 | 0 | 0 | 210.03 | 34.07 | **0.05** |
| **5** | CW | 16 | gelding | 1.04 | 9 | 3 | 0 | 0 | 1 | 0 | 0 | 181.11 | 32.03 | **0.05** |
| **6** | pony | 13 | mare | 1.043 | 9 | 1 | 0 | 0 | 1 | 0 | 0 | 189.57 | 33.95 | **0.05** |
| **7** | pony | 19 | stallion | 1.02 | 8 | 0 | 0 | 0 | 0 | 0 | 0 | 74.96 | 5.07 | **0.13** |
| **8** | Klad | 11 | mare | 1.037 | 9 | 1 | 0 | 0 | 0 | 0 | 0 | 156.31 | 19.10 | **0.07** |
| **9** | Klad | 15 | mare | 1.035 | 8 | 0 | 0 | 0 | 0 | 0 | 0 | 168.34 | 28.10 | **0.05** |
| **10** | Klad | 8 | mare | 1.04 | 9 | 0 | 0 | 0 | 0 | 0 | 0 | 141.41 | 22.11 | **0.06** |
| **11** | CW | 15 | gelding | 1.038 | 9 | 0 | 0 | 0 | 0 | 0 | 0 | 175.44 | 22.43 | **0.07** |
| **12** | Klad | 10 | mare | 1.05 | 9 | 0 | 0 | 0 | 1 | 0 | 0 | 187.73 | 31.33 | **0.05** |
| **13** | Klad | 5 | mare | 1.047 | 8 | 0 | 0 | 0 | 0 | 0 | 0 | 183.22 | 22.72 | **0.07** |
| **14** | Klad | 14 | mare | 1.026 | 9 | 1 | 0 | 0 | 0 | 0 | 0 | 127.26 | 10.77 | **0.10** |
| **15** | Klad | 5 | mare | 1.043 | 8 | 1 | 0 | 0 | 0 | 0 | 0 | 148.43 | 19.12 | **0.07** |
| **16** | CW | 0.2 | stallion | 1.03 | 9 | 0 | 0 | 0 | 0 | 0 | 0 | 561.82 | 24.97 | **0.20** |
| **17** | CW | 0.3 | stallion | 1.02 | 8 | 0 | 0 | 0 | 0 | 0 | 0 | 175.19 | 11.13 | **0.14** |
| **18** | CW | 0.3 | stallion | 1.023 | 8 | 0 | 0 | 0 | 0 | 0 | 0 | 150.33 | 11.57 | **0.11** |
| **19** | Klad | 7 | mare | 1.035 | 9 | 1 | 0 | 0 | 0 | 0 | 0 | 151.55 | 12.34 | **0.11** |
| **20** | Klad | 18 | mare | 1.037 | 8 | 0 | 0 | 0 | 0 | 0 | 0 | 141.79 | 18.58 | **0.07** |
| **21** | Klad | 9 | mare | 1.05 | 9 | 1 | 0 | 0 | 1 | 0 | 0 | 179.58 | 25.42 | **0.06** |
| **22** | Klad | 16 | mare | 1.01 | 7 | 0 | 0 | 0 | 0 | 0 | 0 | 25.26 | 2.10 | **0.11** |
| **23** | Klad | 9 | mare | 1.045 | 9 | 0 | 0 | 0 | 0 | 0 | 0 | 181.65 | 22.74 | **0.07** |
| **24** | Klad | 15 | mare | 1.052 | 9 | 0 | 0 | 0 | 0 | 0 | 0 | 224.52 | 21.65 | **0.09** |
| **25** | Klad | 15 | mare | 1.049 | 9 | 0 | 0 | 0 | 1 | 1 | 0 | 192.61 | 22.63 | **0.08** |
| **26** | CW | 0.1 | mare | 1.009 | 7 | 0 | 0 | 0 | 0 | 0 | 0 | 24.35 | 3.36 | **0.06** |
| **27** | CW | 16 | gelding | 1.045 | 9 | 1 | 0 | 0 | 0 | 0 | 0 | 153.70 | 14.29 | **0.10** |
| **28** | Coldblood | 3 | gelding | 1.044 | 9 | 1 | 0 | 0 | 0 | 0 | 0 | 157.20 | 18.12 | **0.08** |
| **29** | pony | 1 | mare | 1.028 | 8 | 0 | 0 | 0 | 0 | 0 | 0 | 115.99 | 5.41 | **0.19** |
| **30** | Klad | 12 | mare | 1.043 | 9 | 3 | 0 | 0 | 1 | 0 | 0 | 157.28 | 23.71 | **0.06** |
| **31** | Klad | 8 | mare | 1.041 | 9 | 0 | 0 | 0 | 1 | 1 | 0 | 198.74 | 20.30 | **0.09** |
| **32** | CW | 0.2 | stallion | 1.006 | 7 | 0 | 0 | 0 | 0 | 0 | 0 | 21.40 | 2.55 | **0.07** |
| **33** | CW | 0.2 | mare | 1.009 | 6.5 | 0 | 0 | 0 | 0 | 0 | 0 | 31.69 | 5.19 | **0.05** |
| **34** | CW | 19 | gelding | 1.044 | 8 | 1 | 0 | 0 | 1 | 0 | 0 | 177.38 | 44.08 | **0.04** |
| **35** | CW | 0.4 | mare | 1.028 | 8 | 0 | 0 | 0 | 0 | 0 | 0 | 143.21 | 21.40 | **0.06** |
| **36** | CW | 0.1 | mare | 1.004 | 6 | 0 | 0 | 0 | 0 | 0 | 0 | 25.48 | 3.93 | **0.06** |
| **37** | CW | 12 | mare | 1.029 | 9 | 0 | 0 | 0 | 0 | 0 | 0 | 117.60 | 5.72 | **0.18** |
| **38** | CW | 0.4 | stallion | 1.007 | 8 | 0 | 0 | 0 | 0 | 0 | 0 | 51.28 | 3.86 | **0.12** |
| **39** | CW | 0.4 | stallion | 1.011 | 8 | 0 | 0 | 0 | 0 | 0 | 0 | 53.52 | 6.46 | **0.07** |
| **40** | CW | 17 | mare | 1.026 | 9 | 0 | 0 | 0 | 0 | 0 | 0 | 169.73 | 13.57 | **0.11** |
| **41** | CW | 3 | mare | 1.034 | 9 | 1 | 0 | 0 | 0 | 0 | 0 | 204.22 | 18.26 | **0.10** |
| **42** | CW | 8 | mare | 1.034 | 8 | 0 | 0 | 0 | 0 | 0 | 0 | 145.38 | 10.37 | **0.12** |
| **43** | CW | 12 | mare | 1.029 | 7 | 0 | 0 | 0 | 0 | 0 | 0 | 83.58 | 8.61 | **0.09** |
| **44** | CW | 13 | gelding | 1.019 | 8 | 0 | 0 | 0 | 0 | 0 | 0 | 71.34 | 3.52 | **0.18** |
| **45** | CW | 9 | mare | 1.031 | 8 | 0 | 0 | 0 | 0 | 0 | 0 | 160.56 | 12.93 | **0.11** |
| **46** | CW | 10 | mare | 1.048 | 7 | 0 | 0 | 0 | 0 | 0 | 0 | 136.86 | 20.23 | **0.06** |
| **47** | Klad | 19 | mare | 1.045 | 8 | 1 | 0 | 0 | 0 | 0 | 0 | 140.79 | 18.87 | **0.07** |
| **48** | CW | 20 | mare | 1.047 | 7.5 | 0 | 0 | 0 | 0 | 0 | 0 | 133.40 | 20.13 | **0.06** |
| **49** | A1/1 | 1 | mare | 1.043 | 8.5 | 1 | 0 | 0 | 0 | 0 | 0 | 204.60 | 25.65 | **0.07** |
| **50** | A1/1 | 4 | gelding | 1.03 | 8.5 | 1 | 0 | 0 | 0 | 0 | 0 | 159.20 | 19.82 | **0.07** |
| **51** | Haflinger | 21 | gelding | 1.026 | 7 | 0 | 0 | 0 | 0 | 0 | 0 | 90.07 | 10.23 | **0.08** |
| **52** | A1/1 | 3 | mare | 1.033 | 8 | 0 | 0 | 0 | 0 | 0 | 0 | 139.45 | 17.94 | **0.07** |
| **53** | Irish Cob | 6 | mare | 1.046 | 8 | 0 | 0 | 0 | 0 | 0 | 0 | 161.62 | 24.36 | **0.06** |
| **54** | CW | 1.5 | mare | 1.057 | 7.5 | 0 | 0 | 0 | 0 | 0 | 0 | 125.17 | 26.09 | **0.04** |
| **55** | pony | 23 | mare | 1.037 | 9 | 1 | 0 | 0 | 1 | 0 | 0 | 209.57 | 24.74 | **0.07** |
| **56** | CW | 17 | gelding | 1.047 | 8 | 0 | 0 | 0 | 1 | 0 | 0 | 243.68 | 35.77 | **0.06** |
| **57** | Haflinger | 17 | gelding | 1.058 | 8 | 0 | 0 | 0 | 1 | 0 | 0 | 207.01 | 57.32 | **0.03** |
| **58** | CW | 19 | gelding | 1.044 | 8 | 1 | 0 | 0 | 0 | 0 | 0 | 226.14 | 27.71 | **0.07** |
| **59** | CW | 2.5 | mare | 1.052 | 9 | 0 | 0 | 0 | 1 | 0 | 0 | 179.87 | 25.78 | **0.06** |
| **60** | CW | 7 | mare | 1.06 | 8 | 1 | 0 | 0 | 1 | 0 | 0 | 171.71 | 36.43 | **0.04** |
| **61** | CW | 15.5 | mare | 1.04 | 9 | 0 | 0 | 0 | 0 | 0 | 0 | 94.59 | 8.94 | **0.09** |
| **62** | CW | 9 | gelding | 1.033 | 8.5 | 1 | 0 | 0 | 1 | 1 | 0 | 154.35 | 7.9 | **0.17** |
| **63** | CW | 15 | gelding | 1.03 | 8 | 0 | 0 | 0 | 0 | 0 | 0 | 160.01 | 11.9 | **0.12** |
| **64** | CW | 22 | gelding | 1.031 | 8 | 2 | 0 | 0 | 1 | 1 | 0 | 174.08 | 10.6 | **0.14** |
| **65** | A1/1 | 9 | mare | 1.025 | 8 | 0 | 0 | 0 | 0 | 0 | 0 | 160.07 | 13.6 | **0.10** |
| **66** | CW | 11 | mare | 1.045 | 8 | 1 | 0 | 0 | 1 | 0 | 0 | 146.17 | 7.9 | **0.16** |
| **67** | A1/1 | 20 | gelding | 1.03 | 9 | 2 | 0 | 0 | 0 | 0 | 0 | 175.15 | 9.8 | **0.16** |
| **68** | CW | 1.5 | mare | 1.033 | 8 | 2 | 0 | 0 | 0 | 0 | 0 | 202.69 | 24.2 | **0.07** |
| **69** | CW | 1 | mare | 1.039 | 8 | 0 | 0 | 0 | 0 | 0 | 0 | 248.89 | 35.8 | **0.06** |
| **70** | CW | 1.9 | mare | 1.03 | 8 | 2 | 0 | 0 | 1 | 1 | 0 | 243.33 | 31.4 | **0.07** |
| **71** | CW | 16 | mare | 1.04 | 9 | 1 | 0 | 0 | 1 | 0 | 0 | 233.12 | 33.6 | **0.06** |
| **72** | A1/1 | 15 | mare | 1.036 | 8 | 3 | 0 | 0 | 1 | 0 | 0 | 241.69 | 34.6 | **0.06** |
| **73** | CW | 22 | gelding | 1.038 | 8.5 | 2 | 0 | 0 | 0 | 0 | 0 | 235.15 | 36.9 | **0.06** |
| **74** | Haflinger | 5 | gelding | 1.033 | 8 | 0 | 0 | 0 | 0 | 0 | 0 | 205.38 | 16.1 | **0.11** |
| **75** | Haflinger | 5 | gelding | 1.03 | 8 | 0 | 0 | 0 | 0 | 0 | 0 | 183.86 | 23.2 | **0.07** |
| **76** | SW | 9 | gelding | 1.03 | 8 | 1 | 0 | 0 | 0 | 0 | 0 | 211.55 | 18.2 | **0.10** |
| **77** | SW | 10 | gelding | 1.046 | 8 | 2 | 0 | 0 | 0 | 0 | 0 | 193.47 | 21.7 | **0.08** |
| **78** | CW | 21 | gelding | 1.04 | 9 | 1 | 0 | 0 | 1 | 1 | 0 | 182.94 | 26.3 | **0.06** |
| **79** | CW | 24 | gelding | 1.046 | 9 | 1 | 0 | 0 | 1 | 1 | 0 | 205.10 | 16.7 | **0.11** |
| **80** | DW | 12 | mare | 1.033 | 8 | 0 | 0 | 0 | 0 | 0 | 0 | 164.63 | 52.8 | **0.03** |
| **81** | SW | 8 | gelding | 1.044 | 9 | 2 | 0 | 0 | 1 | 1 | 0 | 158.29 | 32.2 | **0.04** |
| **82** | Haflinger | 12 | gelding | 1.036 | 8.5 | 2 | 0 | 0 | 0 | 0 | 0 | 151.77 | 15.6 | **0.09** |
| **83** | A1/1 | 15 | mare | 1.04 | 8.5 | 2 | 0 | 0 | 1 | 1 | 0 | 159.27 | 16.9 | **0.08** |
| **84** | CW | 14 | mare | 1.036 | 8 | 1 | 0 | 0 | 0 | 0 | 0 | 154.29 | 18.1 | **0.08** |
| **85** | CW | 23 | gelding | 1.042 | 9 | 2 | 0 | 0 | 0 | 0 | 0 | 141.86 | 14.8 | **0.09** |
| **86** | Haflinger | 16 | gelding | 1.03 | 8 | 1 | 0 | 0 | 0 | 0 | 0 | 132.28 | 15.8 | **0.07** |
| **87** | Haflinger | 23 | gelding | 1.036 | 8.5 | 0 | 0 | 0 | 0 | 0 | 0 | 136.71 | 16.3 | **0.07** |
| **88** | CW | 20 | gelding | 1.04 | 8.5 | 2 | 0 | 0 | 0 | 0 | 0 | 142.18 | 15.1 | **0.08** |
| **89** | A1/1 | 9 | mare | 1.03 | 8 | 1 | 0 | 0 | 0 | 0 | 0 | 178.04 | 19.5 | **0.08** |
| **90** | A1/1 | 8 | mare | 1.033 | 8 | 0 | 0 | 0 | 0 | 0 | 0 | 176.91 | 20.8 | **0.08** |
| **91** | CW | 7 | mare | 1.034 | 8 | 0 | 0 | 0 | 0 | 0 | 0 | 176.58 | 20.5 | **0.08** |
| **92** | CW | 3 | mare | 1.033 | 8 | 0 | 0 | 0 | 0 | 0 | 0 | 184.33 | 21.9 | **0.07** |
| **93** | CW | 9 | mare | 1.049 | 9 | 1 | 0 | 0 | 1 | 2 | 0 | 174.07 | 22.2 | **0.07** |
| **94** | pony | 7 | mare | 1.04 | 8.5 | 2.5 | 0 | 0 | 0 | 0 | 0 | 157.2 | 27.3 | **0.05** |
| **95** | CW | 9 | gelding | 1.041 | 8.5 | 2 | 0 | 0 | 0 | 1 | 0 | 167.9 | 30.1 | **0.05** |
| **96** | american quarter horse | 11 | gelding | 1.039 | 8 | 1 | 0 | 0 | 0 | 0 | 0 | 155.3 | 36.3 | **0.04** |
| **97** | american paint horse | 10 | gelding | 1.042 | 8.5 | 3 | 0 | 0 | 0 | 0 | 0 | 204.4 | 33.6 | **0.05** |
| **98** | CW | 26 | mare | 1.031 | 8.5 | 2 | 0 | 0 | 0 | 0 | 0 | 129.4 | 13.7 | **0.08** |
| **99** | Polish | 23 | mare | 1.032 | 8.5 | 1 | 0 | 0 | 0 | 0 | 0 | 109.0 | 11.5 | **0.08** |
| **100** | DW | 6 | mare | 1.039 | 9 | 2 | 0 | 0 | 0 | 0 | 0 | 220.5 | 20.8 | **0.09** |
| **101** | CW | 10 | gelding | 1.031 | 9 | 2 | 0 | 0 | 0 | 0 | 0 | 151.3 | 17.6 | **0.08** |
| **102** | Brabant | 2 | gelding | 1.007 | 8 | 0 | 0 | 0 | 0 | 0 | 0 | 50.2 | 2.6 | **0.17** |
| **103** | Oldenburg | 5 | gelding | 1.032 | 9 | 1.5 | 0 | 0 | 0 | 0 | 0 | 144 | 72.6 | **0.02** |
| **104** | BMBH | 13 | gelding | 1.008 | 8 | 0.5 | 0 | 0 | 0 | 0 | 0 | 42 | 3.0 | **0.12** |
| **105** | american quarter horse | 5 | gelding | 1.041 | 9 | 3 | 0 | 0 | 0 | 0 | 0 | 136 | 22.3 | **0.05** |
| **106** | A1/1 | 15 | gelding | 1.036 | 8 | 2 | 0 | 0 | 0 | 0 | 0 | 152 | 26.6 | **0.05** |
| **107** | CW | 11 | mare | 1.014 | 8 | 1 | 0 | 0 | 0 | 0 | 0 | 73 | 6.2 | **0.10** |
| **108** | CW | 11 | mare | 1.039 | 8.5 | 1.5 | 0 | 0 | 0 | 0 | 0 | 167 | 24.5 | **0.06** |
| **109** | BMBH | 21 | gelding | 1.041 | 9 | 2 | 0 | 0 | 0 | 1 | 0 | 199.6 | 22.4 | **0.08** |
| **110** | WelshCob | 12 | mare | 1.048 | 8.5 | 1 | 0 | 0 | 0 | 1 | 0 | 168.1 | 16.5 | **0.09** |
| **111** | CW | 21 | gelding | 1.027 | 8.5 | 1 | 0 | 0 | 0 | 1 | 0 | 96.0 | 14.5 | **0.06** |
| **112** | A1/1 | 20 | mare | 1.037 | 8.5 | 2 | 0 | 0 | 0 | 0 | 0 | 149.8 | 20.2 | **0.07** |
| **113** | A1/1 | 20 | stallion | 1.037 | 8.5 | 2.5 | 0 | 0 | 0 | 0 | 0 | 172.2 | 26.9 | **0.06** |
| **114** | A1/1 | 20 | mare | 1.034 | 8 | 2 | 0 | 0 | 0 | 0 | 0 | 168.8 | 25.7 | **0.06** |
| **115** | Klad | 14 | mare | 1.039 | 8.5 | 2 | 0 | 0 | 0 | 0 | 0 | 147.7 | 14.5 | **0.09** |
| **116** | A1/1 | 13 | stallion | 1.042 | 7.5 | 1 | 0 | 0 | 0 | 0 | 0 | 207.6 | 21.4 | **0.09** |
| **117** | A1/1 | 8 | mare | 1.045 | 9 | 2.5 | 0 | 0 | 0 | 0 | 0 | 156.3 | 26.1 | **0.05** |
| **118** | CW | 20 | stallion | 1.043 | 8 | 2 | 0 | 0 | 0 | 0.5 | 0 | 169.1 | 27.2 | **0.06** |
|  |  |  |  |  |  |  |  |  |  |  |  |  |  |  |
| CW | Czech Warmblood | |  |  |  |  |  |  |  |  |  |  |  |  |
| Klad | Kladruber |  |  |  |  |  |  |  |  |  |  |  |  |  |
| A1/1 | Arabian Horse |  |  |  |  |  |  |  |  |  |  |  |  |  |
| SW | Slovak Warmblood | |  |  |  |  |  |  |  |  |  |  |  |  |
| DW | Dutch Warmblood | |  |  |  |  |  |  |  |  |  |  |  |  |
| BMBH | Bohemian-Moravian Belgian Horse | | |  |  |  |  |  |  |  |  |  |  |  |
